# Road Traffic Accident Traumatic Vehicle Seat Belt Abdominal Wall Hernia

**DOI:** 10.1155/2024/4408980

**Published:** 2024-02-16

**Authors:** Mahmoud R. Manasra, Roua E. Farah, Arein A. Abufara, Bara M. AbuIrayyeh, Rahaf E. Farah, Mohammed A. Maraqa

**Affiliations:** Palestine Polytechnic University College of Medicine and Health Science, Hebron, Palestine, Israel

## Abstract

Traumatic abdominal wall hernia (TAWH) is a rare type of hernia with an incidence of about <1.5%, resulting from blunt abdominal trauma, which leads to an increase in the intra-abdominal pressure and rupture in the abdominal musculature and fascia with herniation of the abdominal organs into the defect. Most TAWH contained either a small bowel (69%) or a large bowel (36%), with 16% containing both. This condition is often not present as an isolated case, as 30% to 60% of the cases are accompanied by other intra-abdominal injuries. The typical manner of presentation is a tender subcutaneous swelling across the abdomen wall with overlaying bruising and ecchymosis. The radiological investigative modality of CT scan has the highest index of diagnosing accompanied intra-abdominal visceral injuries. We present a rare case of a 23-year-old male patient diagnosed with TAWH containing both small bowel and sigmoid colon associated with psoas hematoma caused by a seat belt postroad traffic accident (RTA).

## 1. Introduction

Traumatic abdominal wall hernias (TAWHs) are rare types of hernias that are caused by blunt abdominal injuries. It is seen in 1.5% of blunt abdominal trauma patients. Describe the mechanism by which it occurs as a result of disruption of the abdominal wall muscles and fascia due to increased intra-abdominal pressure [1, 2]. This leads to the inability to maintain abdominal organs and other structures in their usual locations [3]. Most TAWH contained either a small bowel (69%) or a large bowel (36%), with 16% containing both [4] due to its mobility and being an unfixed peritoneal viscera, unlike the large colon (ascending and descending, which are secondarily retroperitoneal organs). These hernias are mostly associated with pelvic or chest injuries [1].

Here, we present a very rare case of small bowel, sigmoid, and greater omentum traumatic abdominal wall hernia in an adult patient caused by a seat belt injury postroad traffic accident. The sigmoid was 12 cm long, and the patient had an incidental, undiagnosed redundant sigmoid colon. The anatomy of the sigmoid was normal with some adhesion, which resulted in a disruption of the left lateral abdominal wall muscles and left rectus abdominis muscle with retraction, creating a large defect from the right anterior superior iliac spine to the left psoas muscle. On physical examination, there was left lower abdomen tenderness with abrasion swelling. Focused assessment with sonography for trauma (FAST) showed minimal abdominal fluid and was negative for pericardium, pelvic fluid, and other solid organ injuries. A chest, abdomen, and pelvic computed tomography (CT) scan with intravenous (IV) contrast was done, demonstrating a traumatic abdominal wall hernia on the left side and a left psoas hematoma. The management of the hernia was operative through the repair of the anterior and posterior fascia using a huge mesh.

This work has been reported in line with SCARE criteria, which are used by authors, journal editors, and reviewers to increase the robustness and transparency of reporting surgical cases [5].

## 2. Case Report

A patient in their 20s with no significant past medical history presented to the ER with abdominal and chest trauma postroad traffic accident (seat belt), complaining of abdominal, neck, and back pain and a headache. The skin was intact. On arrival, the patient was looking well, was not in respiratory distress, and was hemodynamically stable; his vital signs were normal. There is no history of altered levels of consciousness. On chest and physical examination, there is tenderness and no signs of pneumo-haemo thorax; also, there is lower abdominal tenderness with abrasion swelling on the left side (Grey Turner's sign).

**Figure 1:**
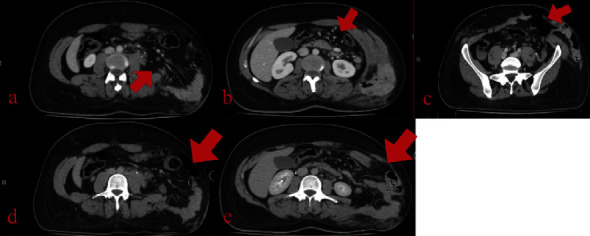
CT abdomen-pelvis imaging: (a–e) axial slices, demonstrating the TAWH containing both small and large bowels.

**Figure 2:**
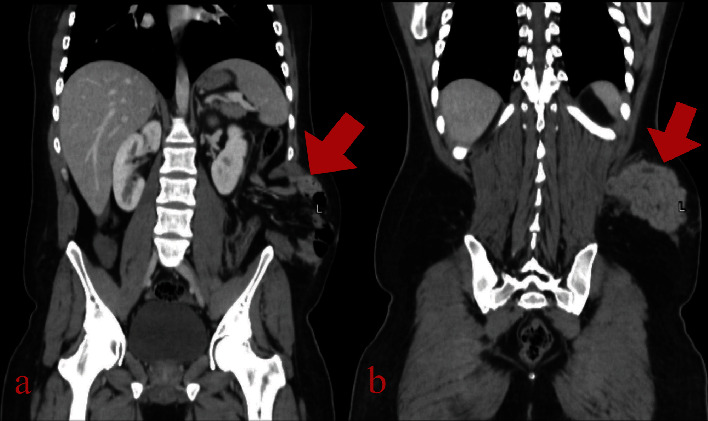
CT abdomen-pelvis imaging: (a, b) coronal slices, demonstrating the TAWH containing both small and large bowels.

**Figure 3:**
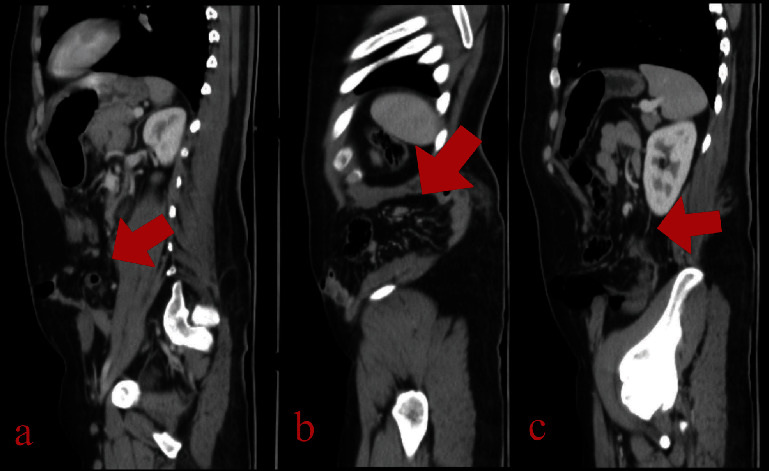
CT abdomen-pelvis imaging: (a–c) lateral slices, demonstrating the TAWH.

According to our centre protocol for road traffic accidents and polytrauma patients, the following are ordered: a focused assessment with sonography for trauma (FAST) in the emergency department showed minimal abdominal fluid and was negative for pericardium and pelvic fluid, as well as chest, abdomen, and pelvic CT scans with IV contrast showed a disruption of the left lateral abdominal wall muscles and the left rectus abdominis muscle with retraction, creating a large defect (up to 15 cm), through which the small bowel, sigmoid, and greater omentum herniate (Figures 1–3), suggesting a traumatic abdominal wall hernia. The left psoas muscle appears bulky (compared to the right) at the level of the 3rd lumbar vertebral body with surrounding fat stranding, suggestive of a left psoas hematoma, and there is no pneumoperitoneum. Little pelvic-free fluid was found. Fractures are linear and nondisplaced in the right transverse processes of the third and fourth vertebrae. The CT scan of the cervical spine is normal. A chest and pelvic X-ray showed left twelfth rib fracture and fractures in the transverse processes of the 3rd and 4th vertebrae.

The general surgery team evaluated the patient and decided on an urgent laparotomy on the next day of admission. The patient underwent an urgent exploratory laparotomy under general anaesthesia through a left paramedian incision with an extension to the left subcostal margin. The patient was fasting, so there is no need for bowl preparation. The intraoperative findings include a large anterior abdominal wall defect (about 25 cm) with crushed muscles and fascia extending from the right anterior superior iliac spine (ASIS) to the left flank, sigmoid serosal injury, a minimal amount of blood in the abdomen, and a mild retroperipheral hematoma. During the procedure, abdominal layers were opened layer by layer. Due to a sigmoid serosal injury with a sigmoid mesentery defect and microperforation, the surgeon decided to do a sigmoidectomy with side-to-side primary anastomosis; the blood supply was also compromised; therefore, the repair is not applicable because the patient was fasting and there is no fecalith peritonitis. We did primary anastomosis instead of colostomy because the defect was in the abdominal wall, and we used a huge Vicryl mesh applied by the sublay technique. Finally, a drain was inserted, and the skin is closed.

The patient remained in the surgical intensive care unit, intubated, and sedated. He was progressing well until postoperative day 7, when he started to have signs of surgical site infection. Body fluid culture showed the growth of extended-spectrum lactamases (ESBLs) producing Klebsiella pneumoniae, sensitive to amikacin. After serial WBCs and CRP follow-up, the patient was discharged home 20 days after trauma, when he was treated for surgical wound infection, and after he was able to mobilise with a walker and tolerate a regular diet.

## 3. Discussion

A rare type of hernia called acute traumatic abdominal wall hernia (TAWH) develops if the abdominal wall is struck by a blunt item at either low or high velocity. Compared to blunt abdominal trauma, which occurs more frequently, TAWHs are less common. According to estimates, TAWH incidence is 1.5% [2, 4]. Handlebar injuries in children and car accidents in adults are the most frequent causes of this injury [6]. The pathophysiology of TAWH involves applying a blunt force to the abdomen over a surface large enough to prevent skin penetration. Tangential forces cause the abdominal wall muscles and fascia to be disrupted as a result of the pressure, which then allows the abdominal viscera to herniate through the defect under the skin [7]. With percentages ranging from 30% to 60%, associated intra-abdominal injuries such as intestine perforations, splenic ruptures, liver avulsions, or pelvic fractures are common [8]. Age, weak abdominal muscles, and pre-existing hernias are risk factors for TAWH [1]. Traumatic abdominal wall hernia (TAWH) can contain either the small bowel or the large bowel; a tiny number of TAWHs can contain both small and large bowels, though this is uncommon [8]. In our case, the abdominal wall hernia contains both the small bowel and the large bowel, in addition to the omentum.

For TAWHs, there is currently no clear classification system [8]. Three different groups of TAWHs can be distinguished. The fascial defect in the first type is significant, and concomitant intra-abdominal injuries are frequent. This type is seen in high-energy traumas. Coexisting intra-abdominal injuries of the second type are uncommon and happen in low-energy traumas. The third type, an intra-abdominal herniation of the colon, is brought on by deceleration trauma [9]. In our case, it combined the first and third types, as it was a high-energy injury that caused hematoma in the psoas muscle, and one of the components of the hernia was a sigmoid colon. Based on the mechanism of injury, Ganchi et al. divided these hernias into two main categories: the “focal” type includes tiny hernias, which are infrequently connected with other injuries, and hernias caused by shearing injuries, as well as other related injuries, more common in the “diffuse” variety [7, 8]. The typical manner of presentation is a tender subcutaneous swelling across the abdominal wall with overlaying bruising and ecchymosis. For a diagnosis, a strong index of suspicion is required. There can be signs of corresponding intra-abdominal damage [7]. For an accurate diagnosis, computed tomography (CT) must be combined with these clinical findings [6].

In our case, CT is helpful to identify the hernia and left-sided psoas muscle injury associated with the fracture of the vertebral transverse process. Traumatic psoas hematoma is a predictor of severe injury and is closely associated with vertebral column fractures. A minority of patients experience extensive blood loss. The psoas hematoma and fracture of the lumbar spine transverse process in this case serve as an illustration of a rare etiology. After using a CT scan in this situation, the diagnosis of iliopsoas injuries was mostly established. Before the advent of CT scans, the diagnosis was challenging and required an autopsy or surgery [10]. The relationship between psoas hematoma and transverse process fractures in the lumbar vertebrae raises the possibility of a cause-and-effect relationship between direct impact trauma to the back or flank. However, a vigorous lateral flexion-extension may partially avulse or disturb the psoas muscle, causing intramuscular bleeding, hematoma, and related abdominal, thoracic, or genitourinary injuries. Transverse process fractures are not typically harmful. Our study's impacted level of vertebral fracture was the right transverse processes of the third and fourth lumbar vertebrae, which is consistent with the findings of other prior research [10].

The danger of concomitant visceral damage may be influenced by the hernia's location. Infraumbilical hernias are less likely to coexist with intra-abdominal injuries, but flank and supraumbilical hernias are more likely to do so. The lower abdominal region, the inguinal region, and the area lateral to the rectus sheath are common sites for TAWH [8]. Early identification and distinction from hematoma are crucial, and a high index of suspicion and awareness of the illness makes treatment easier and improves results. Since rectus sheath hematoma is the primary differential diagnosis, we concur with others that having a high index of suspicion is crucial because the diagnosis is frequently complicated by an associated hematoma [11]. The ability to discriminate between an isolated TAWH and related intra-abdominal injuries makes CT the most reliable diagnostic tool [12]. Ultrasonography can be used for screening; however, CT has been proven to be the most accurate method of diagnosis. It enables accurate visualisation of the abdominal wall and aids in the distinction between a hernia and a rectus sheath haemorrhage [13]. Sonography and CT imaging aid in the quick diagnosis of hematomas. The diffuse involvement of the muscle and a hyperdense lesion in the muscle on CT images are early indicators of hematoma. Performing a CT scan is simple and quick, and the results are helpful for diagnosing, locating, and determining the volume and spatial extent of the lesion. It is also beneficial to drain the hematoma while using CT-guided imaging. However, spontaneous resolution is the injury's natural course of development [10]. The development of the CT scan has made it simple to identify abdominal injuries and decreased the likelihood of complications. It is critical to identify intra-abdominal injuries since bowel incarceration and strangulation can happen in up to 25% of patients [14].

Even though CT is the most frequently used method of diagnosis in trauma patients, if injuries require an emergency response, an exploratory laparotomy will be performed to examine the abdominal wall [1], and in these cases, an additional intra-abdominal injury occurs about 30% of the time [14]. The nature and severity of other lesions, the mechanism and force of the injury, the size of the hernia, and the patient's overall health are all taken into consideration when deciding how to treat a patient [6]. It is widely acknowledged that quick exploration and repair are significantly more effective approaches to managing TAWHs [8]. Delayed exploration and repair may lead to intestinal strangulation and increased tension when the defect is sutured, both of which are linked to higher recurrence rates [15]. Mesh and primary repair have both been used successfully to treat traumatic hernias, despite the fact that the method of treatment is debatable [4]. According to a recent systematic evaluation, tension-free repair is advised to minimise recurrence rates. Due to oedema and haemorrhage during the acute post-traumatic period, this could be challenging to accomplish in a first repair. Mesh use in these circumstances is advised [16]. It has been discovered that using mesh to repair TAWHs results in fewer recurrences than without using mesh. According to a recent study, patients who were treated without the use of mesh experienced up to 70% of recurrences [16]. Mesh repair was advised for all patients with delayed TAWD repair by Liasis et al., but they questioned its utility in emergency situations because of possible contamination. Within the management of traumatic abdominal wall defects, pooled analysis did not reveal any statistically significant differences between mesh versus no mesh repair or between acute versus delayed repair. So, the status of the patient (such as concurrent injuries) should dictate when to have repairs done, ideally with a mesh augmentation [16]. Larger parietal defects can be repaired later because patients with larger defects have a lesser risk of bowel strangulation. This strategy will give the surrounding tissue time to heal, making it safe to employ mesh. However, due to muscle atrophy and retraction, this approach can result in a more challenging repair [17]. Furthermore, up for debate are the ideal incision, the best suture material, and the function of mesh. When dealing with emergencies, midline incisions may be preferred since they make it simpler to explore intra-abdominal injuries and allow for internal defect correction [18]. However, we use the left paramedian incision because the extent of the injury is towards the left flank and the midline incisions will be far from the injury side, which may need another transverse incision. For that reason, our choice was to use the left paramedian incision.
